# The Antimicrobial Peptide Cathelicidin Protects Mice from *Escherichia coli* O157:H7-Mediated Disease

**DOI:** 10.1371/journal.pone.0046476

**Published:** 2012-10-15

**Authors:** Milan Chromek, Ida Arvidsson, Diana Karpman

**Affiliations:** Department of Pediatrics, Clinical Sciences Lund, Lund University, Lund, Sweden; Charité-University Medicine Berlin, Germany

## Abstract

This study investigated the role of the antimicrobial peptide cathelicidin in *Escherichia coli* O157:H7 infection and subsequent renal damage. Mouse and human cathelicidin, CRAMP and LL-37, respectively, killed *E. coli* O157:H7 *in vitro*. Intestines from healthy wild-type (129/SvJ) and cathelicidin-knock-out (*Camp^−/−^*) mice were investigated, showing that cathelicidin-deficient mice had a thinner colonic mucus layer compared with wild-type mice. Wild-type (n = 11) and cathelicidin-knock-out (n = 11) mice were inoculated with *E. coli* O157:H7. Cathelicidin-deficient animals exhibited higher fecal counts of *E. coli* O157:H7 and bacteria penetrated the mucus forming attaching-and-effacing lesions to a much higher extent than in wild-type animals. Cathelicidin knock-out mice developed symptoms (9/11) as well as anemia, thrombocytopenia and extensive renal tubular damage while all cathelicidin-producing mice remained asymptomatic with normal laboratory findings. When injected with Shiga toxin intraperitoneally, both murine strains developed the same degree of renal tubular damage and clinical disease indicating that differences in sensitivity to infection between the murine strains were related to the initial intestinal response. In conclusion, cathelicidin substantially influenced the antimicrobial barrier in the mouse colon mucosa. Cathelicidin deficiency lead to increased susceptibility to *E. coli* O157:H7 infection and subsequent renal damage. Administration of cathelicidin or stimulation of endogenous production may prove to be novel treatments for *E. coli* O157:H7-induced hemolytic uremic syndrome.

## Introduction

In humans, infection with enterohemorrhagic *Escherichia coli* (EHEC) often causes diarrhea. Many EHEC serotypes have been isolated but the most prevalent is *E. coli* O157:H7 [1]. EHEC is non-invasive and does not cause bacteremia. However, EHEC adheres intimately to the intestinal mucosa and forms attaching and effacing lesions [2]. These lesions are characterized by close contact between the bacteria and epithelial cells and effacement of intestinal microvilli [3]. The intimate attachment and destruction of the intestinal epithelium enable secretion of bacterial virulence factors into the systemic circulation. Amongst them, Shiga toxin (Stx) is considered to play a pivotal role in the subsequent tissue damage [4]. The toxin secreted in the intestine circulates bound to blood cells (neutrophils, monocytes and platelets) before reaching its target organs [5]. The location of Stx receptors in the kidney and brain is predictive of the sites of action of Shiga toxin [4]. The toxin can cause apoptosis or generate an inflammatory response in the affected cells [6]. The major complication of EHEC infection is hemolytic uremic syndrome (HUS), which occurs in up to 15% of patients with EHEC infection. HUS is characterized by non-immune microangiopathic hemolytic anemia, thrombocytopenia and acute renal failure and is a major cause of acute kidney injury in children [7].

Antimicrobial peptides are distributed throughout the animal and plant kingdoms suggesting that they have served a fundamental role in the successful evolution of multi-cellular organisms protecting them against prokaryotes [8]. Two main families of antimicrobial peptides in mammals, the defensins and the cathelicidins, are expressed in immune cells and at epithelial surfaces. There are several defensins but only one cathelicidin in humans and mice [8]. The cathelicidin proform is processed to the mature bioactive peptide, which is called LL-37 in humans [9] and CRAMP (cathelin-related antimicrobial peptide) in mice [10]. LL-37 and CRAMP are highly homologous, both are amphipathic, α-helical peptides, which preferentially bind to negatively charged groups on the outer leaflet of the bacterial membrane thus inducing damage. Cathelicidins also inhibit the formation of bacterial biofilm [11], [12] and modulate multiple processes related to both innate and adaptive immunity [13]. Cathelicidin knock-out mouse models have confirmed the importance of cathelicidin in protection of the skin [14] as well as the urinary [15] and gastrointestinal tracts [16] from bacterial infections.

In the mouse and human colon cathelicidin is expressed in epithelial cells and macrophages [16], [17], [18]. It has been suggested that bacterial pathogens must overcome the mucus-antimicrobial peptide layer in order to colonize the intestine [19].

The aim of this study was to investigate the effects of cathelicidin on *E. coli* O157:H7 infection. To this end the *in vitro* effect of cathelicidin on *E. coli* O157:H7 survival was assayed. The intestinal mucosal layer was investigated using cathelicidin wild-type and deficient mice and these mice were further infected with *E. coli* O157:H7 to determine if cathelicidin exerted a protective effect against infection.

## Materials and Methods

### Ethics statement

All animal experiments were approved by the Animal Ethics Committee of Lund University in accordance to guidelines of the Swedish National Board of Agriculture and the EU directive for the protection of animals used for scientific purposes.

### Bacteria

*E. coli* O157:H7, both Stx2-producing (86–24) and non-producing (87–23) strains, were isolated in November 1986 during the Walla Walla Washington State outbreak of hemorrhagic colitis and HUS [20], and were kindly provided by A. D. O'Brien (Uniformed Services University of the Health Sciences, Bethseda, MD). The strains were previously genotypically and phenotypically characterized, found to be isogenic, and differed only in the production of Stx2 [21], [22]. Spontaneous streptomycin-resistant derivatives of these strains were isolated as previously described [23].

### Bacterial sensitivity assay

The minimal inhibitory concentrations (MIC) and 50% bactericidal concentrations (IC_50_) were determined using *E. coli* O157:H7 and the synthetic murine peptide CRAMP consisting of 39 amino acids, as well as the synthetic human peptide LL-37, consisting of 37 amino acids, both from Innovagen, Lund, Sweden, by a microtiter plate method (modified according to The Swedish Reference Group for Antibiotics- subcommittee on methodology, www.srga.org) as described before [15]. Briefly, bacteria were grown overnight on LB agar plates and resuspended in LB broth at a concentration of 10^5^ bacteria/ml. Bacterial suspensions (90 µl) were grown in the presence of different concentrations of the peptide (10 µl). The final peptide concentrations ranged from 1.25 µM to 20 µM. The solutions were incubated at 37°C for 30 min and for 16 h. IC_50_ after 30 min was analyzed by plating serial dilutions of the bacteria-peptide solutions on LB plates. The MIC was determined after 16 h as the lowest concentration yielding no visible bacterial growth.

### Mice

129/SvJ wild-type and CRAMP-deficient (*Camp^−/−^)* mice, generated on the same background [14], were kindly provided by B. Agerberth, Karolinska Institute, Stockholm, Sweden. For the infection experiments, both male and female mice were used at 8 to 33 weeks of age. Mice used for Stx2 injection experiments were up to 1 year old.

### Analysis of the thickness and amount of intestinal mucus

Uninfected wild-type and *Camp^−/−^* mice were sacrificed by cervical dislocation. One-cm-long segments of colon together with fecal content were carefully collected and dipped in a water-free Methanol-Carnoy's fixative (60% dry methanol, 30% chloroform and 10% acetic acid) [24] and fixed overnight. The tissues were then washed in methanol before embedding in paraffin, and 4 µm sections were placed on glass slides. Slides underwent deparaffinization with xylene, rehydration with 99.5% and 95% ethanol. The slides were then stained according to the standard Periodic acid-Schiff method and assessed by light microscope. The thickness of the inner stratified mucus layer was measured using a digital camera and AxioVision software (Carl Zeiss). To quantify the mucus content, an established colorimetric method with Alcian blue was used with minor modifications [25]. Briefly, immediately after euthanasia, 1-cm-long segments of colon were collected, opened, everted, weighed (without fecal content) and soaked for 2 h in a 0.1% solution of Alcian blue dissolved in 0.16 M sucrose and buffered with 0.05 M sodium acetate, pH 5.8. Afterwards, the segments were washed twice in a 0.25 M sucrose solution for 15 min to extract uncomplexed dye. Segments were then placed in a 10 g/L docusate sodium salt solution overnight at room temperature to extract the absorbed dye from the tissue. The extracted dye corresponded to intestinal mucus. After centrifugation at 700×g for 5 min, 100 µl of the supernatant was transferred to a 96-well plate (in duplicate) together with a standard curve from an Alcian blue solution. Optical density was measured at 630 nm and results were expressed as the amount of absorbed dye per mg of colonic tissue.

### Mouse model of *E. coli* O157:H7-mediated disease

Animal experiments were performed as described before with modifications [23]. An initial experiment was performed with intact bacterial flora. In the remaining experiments mice were treated with streptomycin sulfate (5 g/L) in drinking water for 24 h before inoculation and during the entire experiment to reduce normal flora and to enable colonization by the streptomycin-resistant strain.

Bacterial strains were grown overnight at 37°C in LB broth supplemented with 50 µg/ml streptomycin. After centrifugation, the pellet was washed in sterile PBS and resuspended in a solution of 20% (w/v) sucrose and 10% (w/v) NaHCO_3_ in sterile water at a concentration of 10^9^ colony forming units/ml. The bacterial concentration was confirmed by plating serial dilutions of the bacterial suspension on LB agar plates.

The mice were fasted for 16 h and then pipette-fed with 100 µl of prepared bacterial suspension corresponding to 10^8^ CFU/mouse. Control animals received sucrose/NaHCO_3_ solution without bacteria. After bacterial challenge food was reintroduced and provided *ad libitum*. Weight was monitored daily and mice were monitored three to four times per day. Feces were collected on days 1, 3, 6, 9, 12, weighed, homogenized, suspended in 1 ml PBS and, after serial dilutions in PBS, plated on LB agar plates supplemented with streptomycin (50 µg/ml) to follow bacterial colonization in feces. The identity of bacterial colonies on plates was confirmed by the O157LPS latex agglutination test (Oxoid, Basingstoke, UK). After 14 days or when evident signs of disease were observed (ruffled fur, hunched posture, decreased activity or neurological signs), blood was sampled under isoflurane anesthesia. For assay of hemoglobin a drop of blood from the tail tip was aspirated. For platelet counts, blood smear and creatinine levels a larger amount of blood (300–900 µl) was collected into a citrated syringe by heart puncture. The mice were then sacrificed by cervical dislocation, and kidneys as well as colon segments were collected for further analysis. In certain experiments tail blood was sampled before bacterial inoculation for the assay of hemoglobin and platelet counts.

Hemoglobin levels were analyzed directly from tail tip blood by a spectrophotometer. Levels were adjusted for body weight change in individual mice to account for hemoconcentration due to dehydration. A part of the citrated blood was treated with paraformaldehyde to a final concentration of 0.5% to eradicate possible contamination with bacteria and enable transfer of the sample. This was done for blood smears and platelet-rich plasma. Platelet-rich plasma was achieved by diluting citrated blood in PBS (1∶3) followed by centrifugation at 180×g for 60 seconds. Platelets were counted using a hemocytometer. To obtain platelet-poor plasma for creatinine measurements, blood was first centrifuged at 1000×g for 10 minutes and plasma samples were filtered through 0.2 µm pore filters (Whatman, Dessel, Germany) and stored at −80°C until analyzed. Creatinine levels were measured using a colorimetric kit (Cayman Chemical, Ann Arbor, MI) following the manufacturer's instructions.

### Intraperitoneal injection of Stx2

Purified Stx2 (obtained from C Thorpe, Phoenix Lab, Tufts Medical Center, Boston, MA) was diluted in PBS and injected intraperitoneally in 129/SvJ and *Camp^−/−^* mice at a dose 285 pg/g weight as described before [23]. Mice were then followed for 10 days as described above and sacrificed by cervical dislocation.

### Immunofluorescence analysis for O157LPS and F-actin

Sections of one-cm-long segments of the colon including fecal content were carefully collected and dipped in a water-free Methanol-Carnoy's fixative overnight, washed in methanol, embedded in paraffin, sectioned (4 µm) onto positively charged glass slides, deparaffinized, rehydrated and washed with tap water. Antigen retrieval was performed by boiling the sections in 10 mM citrate buffer pH 6.0 for 15 min in a microwave oven. Sections were blocked using 10% normal rabbit serum in PBS for 1 h at 37°C and incubated with 14 µg/mL polyclonal goat anti-O157LPS antibody (Fitzgerald Industries, Acton, MA, USA) and with 5 µg/mL phalloidin TRITC (Sigma-Aldrich, Schnelldorf, Germany) in PBS for 1 hr at room temperature in a humidity chamber. Thereafter, after washing in PBS, the slides were incubated with FITC-conjugated rabbit anti-goat antibody (Dako, Glostrup, Denmark) 1∶600 for 1 h at room temperature. Specificity of immunolabelling was controlled by using normal goat IgG instead of primary anti-O157LPS antibody, as well as by analysis of uninfected animals. Slides were washed in PBS and mounted with Vectashield DAPI (Vector Laboratories, Burlingame, CA, USA) mounting medium. Images were acquired with a fluorescent microscope.

### Histological analysis of kidneys

After 14 days from bacterial inoculation or when evident signs of disease were observed, mice were sacrificed by cervical dislocation. The kidneys were removed and immediately dipped into 4% paraformaldehyde solution in PBS for one to three days. The tissues were then embedded in paraffin, sectioned and 4 µm sections were placed on glass slides. Slides underwent deparaffinization and staining by hematoxylin and eosin.

### Statistical analyses

Data are presented as dot plots and medians (for data, which did not pass the criteria for normal distribution) or means (normally distributed data). Mann-Whitney U test (not-normally distributed data) or t-test (normally distributed data) were used to compare two groups. Morbidity is depicted as a survival curve and analyzed by log-rank test. For repeated measurements, two-way ANOVA was used. P values<0.05 were considered statistically significant.

## Results

### Cathelicidin kills *E. coli* O157:H7 *in vitro*

*E. coli* O157:H7 strain 86–24 was incubated with synthetic mouse cathelicidin CRAMP and tested for the peptide concentration killing 50% of the bacteria after 30 min (IC_50_) and minimal inhibitory concentration (MIC) after 16 hours. The IC_50_ was 1 µM, corresponding to 4.4 µg/ml CRAMP and the MIC of the peptide was between 5–10 µM, corresponding to 22–44 µg/ml CRAMP. Similar results were obtained using synthetic human cathelicidin LL-37 with IC_50_ of 1 µM, corresponding to 4.5 µg/ml LL-37, and a MIC of 5–10 µM, corresponding to 22.5–45 µg/ml LL-37.

### CRAMP-deficient mice have a thinner layer of mucus in the colon

**Figure 1:**
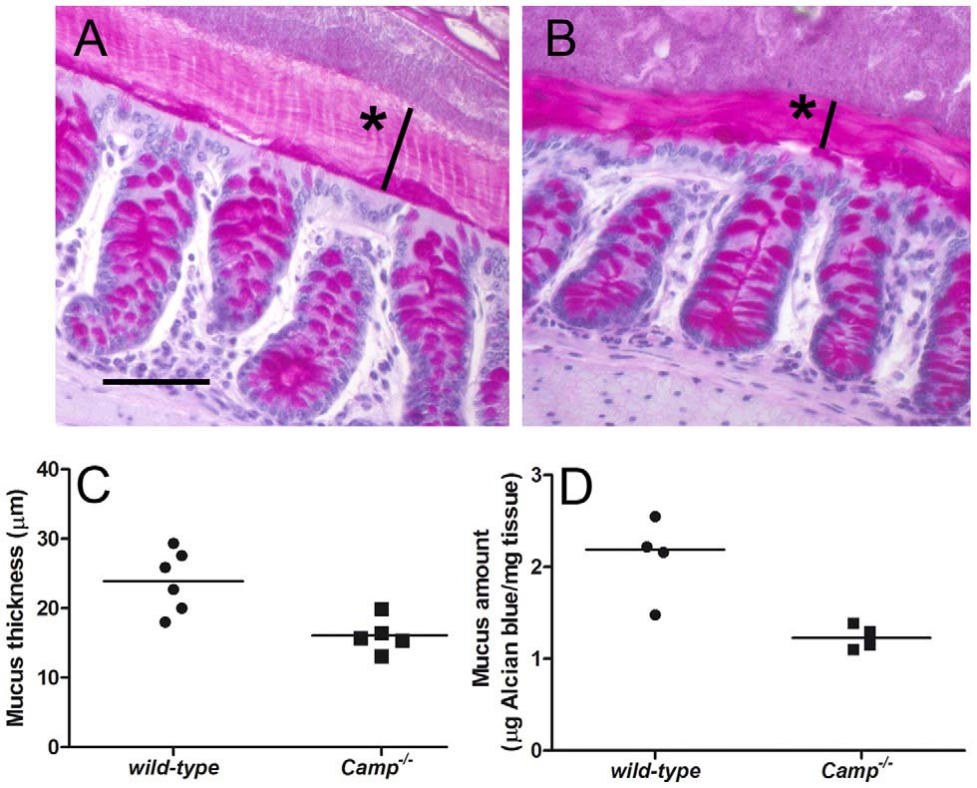
CRAMP-deficient mice have a thinner layer of mucus in the colon. (**A** and **B**) Mouse colon tissues from a representative colonic segment taken from 129/SvJ (**A**, wild-type) and *Camp^−/−^* (**B**) mice, fixed by water-free Methanol-Carnoy's fixative, stained with PAS and visualized by light microscopy. Stars indicate thickness of the inner mucus layer. Scale bar indicates 25 µm. (**C**) The thickness of the inner stratified layer of colonic mucus in µm. The individual values and means are displayed for each group of mice (N = 6 and 5, respectively). The inner mucus layer in *Camp^−/−^* mice was thinner by 32.7% (p<0.01) (**D**) The total amount of colonic mucus expressed as the amount of Alcian blue bound to mg of colonic tissue. The individual values and medians are shown (N = 4 in each group). The amount of mucus in *Camp^−/−^* mice was lower by 43.8% compared with the wild-type animals (p<0.05).

Cathelicidin has been suggested to stimulate mucin production in the gut [26]. We therefore analyzed the thickness and amount of colonic mucus in a healthy uninfected state, in mice with and without cathelicidin. The inner stratified layer of mucus is more constant and easier to visualize [24]. The thickness of the inner stratified mucus was measured in sections stained with PAS and analyzed by light microscopy (Figure 1A and B). The inner mucus layer in cathelicidin-deficient mice (N = 5) was thinner by 32.7% as compared with the mucus layer of wild-type animals (N = 6, p<0.01, Figure 1C). In addition, the total amount of mucus was measured by Alcian blue binding in colon segments from 4 mice in each group. The total amount of colonic mucus was lower by 43.8% in CRAMP knock-out mice as compared with the wild-type animals (p<0.05, Figure 1D).

### CRAMP deficiency facilitates colonization with *E. coli* O157:H7

**Figure 2:**
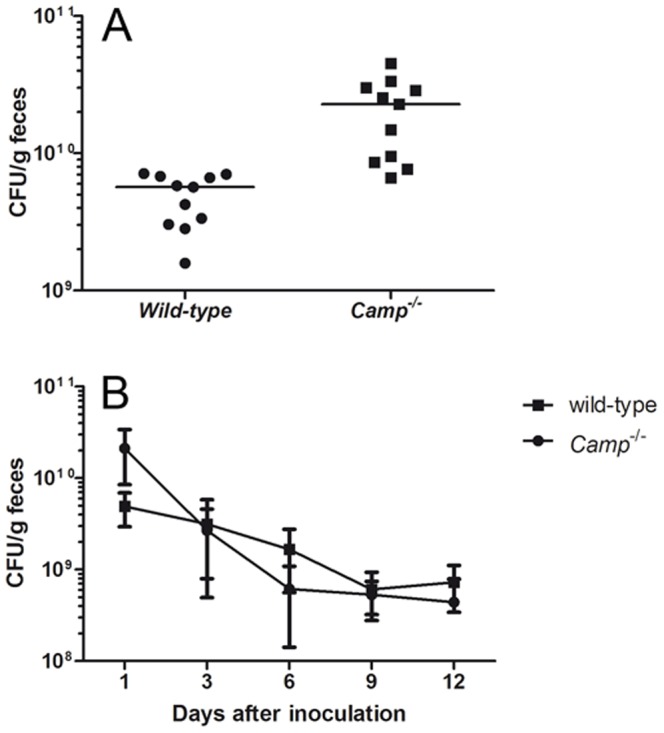
CRAMP-deficient mice are more easily colonized with *E. coli* O157:H7. (**A**) Bacterial colonization one day after inoculation with *E. coli* O157:H7 expressed as CFU/g feces. Colonization of cathelicidin-deficient animals was significantly higher, p<0.001. Data show individual values and means from three independent experiments, N = 11 in each group. (**B**) Dynamics of bacterial colonization up to 12 days after inoculation with *E. coli* O157:H7 expressed as CFU/g feces. From the 3rd day of the experiment, the degree of colonization of CRAMP-deficient animals decreased and reached the levels observed in wild-type mice. Data show means and SD from three independent experiments, N = 11 mice in each group.

In a preliminary experiment, five CRAMP-producing (129/SvJ) and five CRAMP-deficient mice were inoculated orally with *E. coli* O157:H7 10^8^ CFU/mouse without antibiotic treatment. These mice were not successfully colonized and did not develop any signs of disease. Therefore, in order to reduce the normal intestinal flora and thus enable better colonization 129/SvJ (N = 11) and *Camp^−/−^* (N = 11) mice were, in the following three experiments, treated with streptomycin for 24 hr before challenge with *E. coli* O157:H7 10^8^ CFU/mouse as previously described [23]. Antibiotic treatment was maintained throughout the experiments. Viable bacterial counts were performed in fecal samples. One day after inoculation the wild-type mice had 4.9×10^9^ CFU *E. coli* O157:H7/g feces (mean, SD 1.98×10^9^). Colonization of cathelicidin-deficient animals was approximately four-fold higher, 2.1×10^10^ CFU of *E. coli* O157:H7/g feces, (mean, SD 1.27×10^10^, p<0.001, Figure 2A). From the 3rd day of the experiment, the degree of colonization of CRAMP-deficient animals decreased and reached the levels observed in wild-type mice (Figure 2B).

### *E. coli* O157:H7 penetrate the mucus of CRAMP-deficient mice and form attaching and effacing lesions

**Figure 3:**
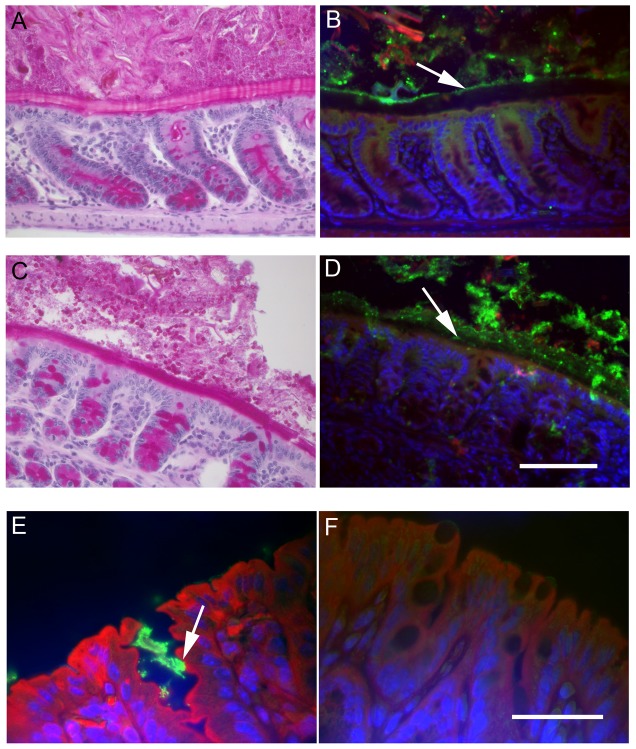
*E. coli* O157:H7 penetrate the mucus of CRAMP-deficient mice and form attaching and effacing lesions. (**A**–**D**) (**A** and **C**) PAS-stained and (**B** and **D**) corresponding immunofluorescence sections from mouse colon of wild-type (**A** and **B**) and CRAMP-deficient mice (**C** and **D**) inoculated with *E. coli* O157:H7. (**B**) In CRAMP-producing wild-type mice, *E. coli* O157:H7 (green) was localized in the intestinal content and did not penetrate the inner stratified mucus layer (arrow). (**C**) In CRAMP-deficient mice the inner mucus layer was thinner and less homogenous and (**D**) *E. coli* O157:H7 could be seen within the inner mucus layer and or even in close proximity to the intestinal epithelial cells (arrow). Green indicates O157LPS (antibody), red F-actin (phalloidin) and blue cell nuclei (DAPI). The scale bar indicates 50 µm (**E** and **F**) Immunofluorescence staining of colonic segments from a CRAMP-deficient mouse infected with *E. coli* O157:H7 (**E**) and from a CRAMP-deficient non-infected mouse (**F**). In CRAMP-deficient mice, attaching and effacing lesions (arrow) were observed with bacteria adhered to intestinal epithelial cells (green) and actin polymerization (red). Green indicates O157LPS (antibody), red F-actin (phalloidin) and blue cell nuclei (DAPI). The scale bar indicates 25 µm.

In order to analyze the interaction between *E. coli* O157:H7, intestinal mucus and intestinal epithelial cells *in vivo*, segments of mouse colon prepared in Methanol-Carnoy's fixative were stained with fluorescent-labeled antibody against O157LPS as well as fluorescent-labeled phalloidin, which preferentially binds to F-actin. Polymerization of actin is one of the processes typical for attaching and effacing lesions of adherent bacterial pathogens such as *E. coli* O157:H7 [27]. In CRAMP-producing wild-type mice, *E. coli* O157:H7 were only localized in the intestinal content and did not reach the epithelium (Figure 3A and B), indicating that the inner stratified mucus layer effectively protected intestinal epithelial cells from bacterial attachment. The stratified mucus layer of the CRAMP-deficient animals, on the other hand, was thinner and non-homogenous, and *E. coli* O157:H7 were localized in the inner mucus layer or even in close proximity to the intestinal epithelial cells (Figure 3C and D) where typical attaching and effacing lesions with F-actin were observed in 5/5 analyzed mice (Figure 3E). Controls consisted of uninfected mice as well as intestines from infected mice stained with the control antibody. These controls did not exhibit bacterial staining (as shown in Figure 3F for the uninfected control).

### CRAMP-deficient mice develop HUS-like disease after inoculation with *E. coli* O157:H7

**Figure 4:**
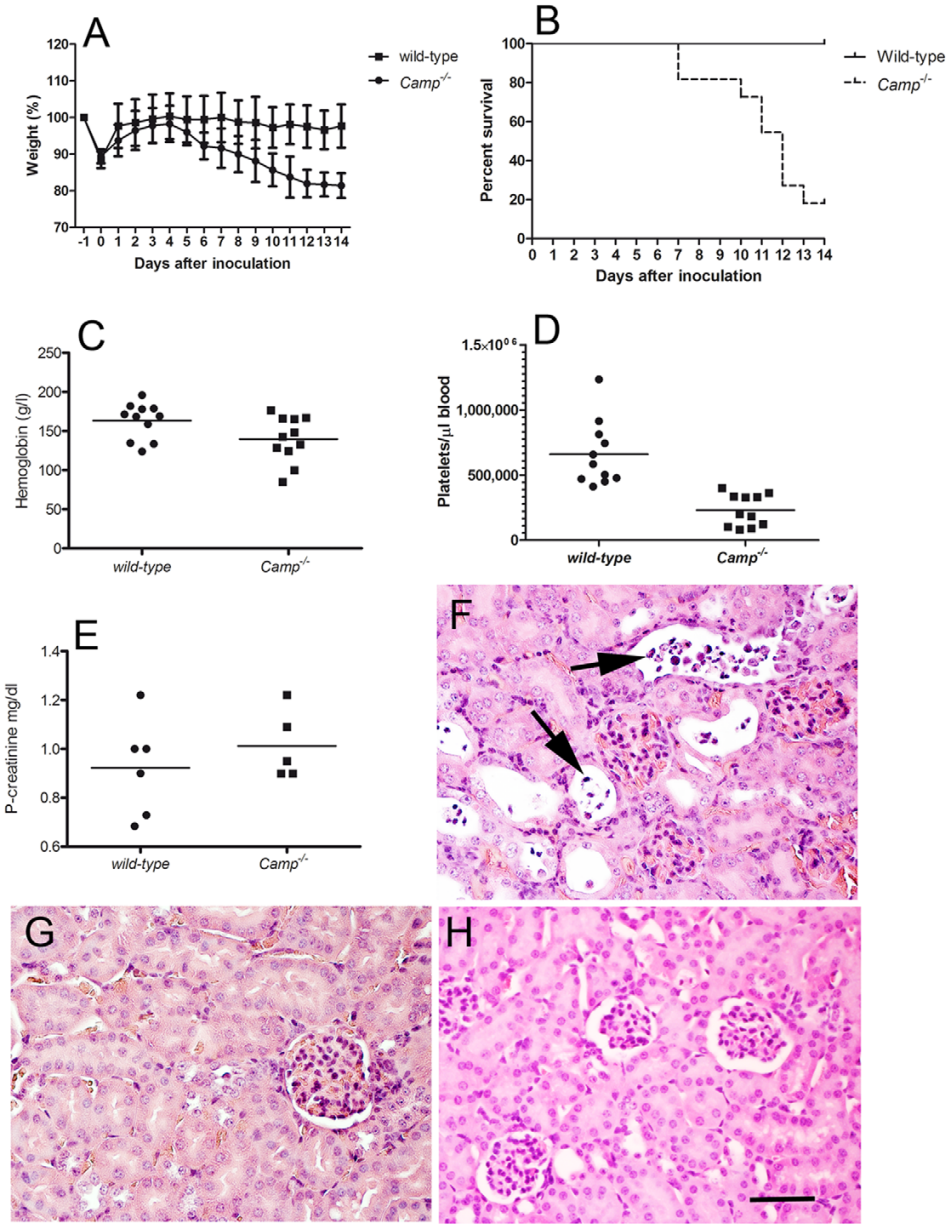
CRAMP-deficient mice develop symptomatic disease after inoculation with *E. coli* O157:H7. (**A**) Relative weight of wild-type and CRAMP-deficient (*Camp^−/−^*) animals after inoculation with *E. coli* O157:H7. Weight is expressed in relation to weight at the beginning of the experiment (before fasting) which was set to be 100%. Data show means and SD from three independent experiments, with a total of N = 11 mice in each group. (**B**) Morbidity of wild-type and *Camp^−/−^* mice depicted as a survival curve. Data represent a summary of three independent experiments and 11 mice in each group. Of the knock-out animals (9/11, 82%) gradually developed clinical signs of disease while all wild-type animals remained asymptomatic (p<0.001). (**C**) Hemoglobin (g/l) and (**D**) platelet counts (/µl) 14 days after *E. coli* O157:H7 inoculation or when evident signs of disease were observed. Means and individual values are presented (N = 11 in each group, three independent experiments, p<0.05 and p<0.0001, respectively). (**E**) Plasma creatinine (mg/dl) from the same mice as in (**C**) and (**D**) (p>0.05). (**F**) Renal cortex from a representative *Camp^−/−^* mouse. Desquamation of tubular epithelial cells with typical apoptotic features such as chromatin condensation, cell shrinkage and membrane blebbing (arrows). (**G**) Renal cortex from a representative infected wild-type mouse showing normal histology. (**H**) Normal renal cortex from a representative *Camp^−/−^* mouse treated with sterile sucrose/NaHCO_3_ solution instead of bacteria. Sections are stained with hematoxylin and eosin. The scale bar indicates 50 µm.

*E. coli* O157:H7 inoculated CRAMP-producing (129/SvJ) and CRAMP-deficient mice were followed for 14 days. While the wild-type animals remained asymptomatic during the entire observation period, CRAMP-deficient mice started losing weight on day 5 post-inoculation (Figure 4A). From the 7th day after the inoculation 9/11 knock-out animals developed clinical signs of disease manifesting as ruffled fur, hunched posture, decreased activity and lethargy. The majority of *Camp^−/−^* animals became ill between day 10 and 13 (survival p<0.001, Figure 4B). Analyses of the wild-type animals showed normal levels of hemoglobin, platelet counts and plasma creatinine. In contrast to wild-type animals, *Camp^−/−^* mice developed anemia (p<0.05, Figure 4C) and thrombocytopenia (p<0.0001, Figure 4D). Fragmented red blood cells were not visualized in any of the animals. Unfortunately, sufficient samples for analysis of plasma creatinine were not available from all mice. The difference in the analyzed samples (N = 6 and N = 5, respectively) was not statistically significant (p>0.5, Figure 4E). However, microscopic analysis of mouse kidneys revealed in all CRAMP-knock-out mice (including the two asymptomatic mice) extensive renal tubular damage with desquamation of tubular epithelial cells and typical apoptotic features such as chromatin condensation, cell shrinkage and membrane blebbing (Figure 4F). Minimal tubular damage was observed in the kidneys from 3/11 of the wild-type animals (Figure 4G). No glomerular pathology was observed in any of the groups.

As controls four wild-type and three *Camp^−/−^* mice were treated with sucrose/NaHCO_3_ solution instead of bacteria. The control mice were naturally not colonized with *E. coli* O157:H7 and did not develop any symptoms, laboratory or histopathologic signs of disease (Figure 4H).

To address the role of Stx for development of symptoms, wild-type and *Camp^−/−^* mice were treated with streptomycin and inoculated with Stx2-producing *E. coli* O157:H7 (strain 86-24, N = 4 wild-type and N = 4 *Camp^−/−^* mice) and Stx non-producing (strain 87-23, N = 1 and 2, respectively). This control experiment was included in one of three independent experiments in which mice infected with the Stx2-producing strain 86-24 were monitored, as described above. The two bacterial strains colonized the intestinal mucosa in both mouse strains but only the Stx2-producing *E. coli* O157:H7 induced disease in cathelicidin-deficient mice (4/4). The three mice infected with the Stx2-negative *E. coli* O157:H7 strain remained asymptomatic, both cathelicidin-producing (n = 1) and cathelicidin-knock-out (n = 2) animals.

To test the sensitivity of wild-type (N = 4) and *Camp^−/−^* (N = 3) mice for the major *E. coli* O157:H7 virulence factor Stx, purified Stx2, was injected intraperitoneally at 285 pg/g weight which was earlier shown to be highly toxic for C57BL/6 mice [23]. In both wild-type and *Camp^−/−^* mice intraperitoneal Stx2 injection led to the development of clinical disease in only one mouse in each group (1/4 and 1/3, both on day 3 after injection). The rest of the mice remained asymptomatic and without significant differences in weight. Laboratory analysis showed significantly decreased hemoglobin after injection with Stx2 in both groups of animals (p<0.001, comparison of levels before and after injection in all mice), but no clear differences between the two groups of mice or between the mice with symptoms and the asymptomatic mice. After intraperitoneal injection of Stx2, increased levels of creatinine in plasma (mean 1.9 mg/dl, SD 0.9) and normal platelet counts were measured. Plasma creatinine and platelet counts did not differ between the wild-type and *Camp^−/−^* mice with a reservation due to the limited number of mice. Histopathological analyses of the kidneys revealed renal tubular damage similar to that visualized in mice inoculated with *E. coli* O157:H7 in both groups of animals regardless of symptoms and without any distinct differences between the groups.

## Discussion

In this study we show that the antimicrobial peptide cathelicidin significantly influences both the mechanical and the antimicrobial barrier in the gut. Deficiency of cathelicidin leads to high susceptibility to pathogenic *E. coli* O157:H7 with development of life-threatening disease.

Every human being carries an abundance of bacteria on different epithelial surfaces, mainly in the gut [28]. Intestinal bacteria play important functions especially in metabolism and immunity [29] but can also become pathogens causing disease. Effective and delicate protective mechanisms are therefore needed. The mucosal surfaces are protected from invading microorganisms by biological, mechanical and chemical barriers.

The *biological barrier* consists of normal flora, i.e. commensal bacteria which modulate the innate immunity and inhibit the growth of other microorganisms. Multicellular organisms and normal flora have evolved together for thousands of years and influence each other by different mechanisms. For example, commensal bacteria produce short fatty acids, which can easily diffuse through the mucus layer and serve as an important source of energy for colonocytes [30]. Moreover, short fatty acids stimulate epithelial cells in the colon and affect their differentiation and production of antimicrobial peptides [31]. Specific cocktails of antimicrobial peptides create an environment that is hostile to certain microorganisms. Mucins produced by goblet cells in the colon, on the other hand, may serve as receptors for bacterial adhesins [32] and give signals that welcome bacteria to stay at the epithelial surface [33].

Our experiments confirmed the paramount importance of the biological barrier for the protection against the pathogenic *E. coli* O157:H7. To establish a successful colonization with this microorganism we first needed to reduce normal flora by antibiotic treatment. Pathogenic bacteria were otherwise not able to colonize the gastrointestinal tract and animals remained healthy.

*The mechanical barrier* in the colon is formed by a layer of mucus, which is continuous, relatively thick (at least 150 µm in the mouse) and consists of an inner stratified firm layer and an outer loose layer [24]. The importance of mucus has been neglected for a long time due to technical reasons. The classical ways of fixing and preserving biopsy or necropsy specimens involve the use of cross-linking fixatives such as formaldehyde or paraformaldehyde. Such fixatives cause the mucus layer to collapse into a very thin film and bacteria in such preparations seem to be in close proximity with epithelial cells. Careful use of denaturating water-free fixatives, on the other hand, revealed that gut bacteria are separated from the epithelial cell barrier by a thick and continuous layer of mucus, free from bacteria [34]. Our experiments confirmed not only the presence but also the importance of the thickness and quality of mucus for the protection against the adhering pathogen *E. coli* O157:H7. The antimicrobial peptide cathelicidin stimulates production of mucins [26], [35]. Therefore, not surprisingly, cathelicidin-deficient mice had a thinner and non-homogenous mucus layer compared with cathelicidin-producing animals. As a result, pathogenic *E. coli* bacteria were able to penetrate the mucus, attach to the epithelial cells and form attaching and effacing lesions as visualized by immunofluorescence.

The third line of protection is *chemical protection*. Epithelial cells in the gastrointestinal tract produce a number of antimicrobial proteins and peptides such as α- and β-defensins, lysozyme, secretory phospholipase A2, elafin, bactericidal/permeability-increasing protein and cathelicidin [36] making the environment in close proximity to epithelial cells hostile to certain bacteria. It has been suggested that secreted antimicrobial peptides are retained by the intestinal mucus and thereby provide a combined physical and chemical (antimicrobial) barrier against invading pathogens [37]. Animal models have confirmed the relevance of defensins in gastrointestinal mucosal defence. Mice lacking matrilysin (MMP7), the protease that processes pro α-defensin into active compounds, have impaired ability to eliminate non-pathogenic *E. coli* and *Salmonella typhimurium* [38]. In addition, mice that express human defensin 5 have increased resistance to the latter bacterium [39].

In our experiments, we focused on the antimicrobial peptide cathelicidin. Cathelicidin is expressed within the epithelial cells and macrophages of normal human and mouse colon [16], [17], [18] and it was shown to significantly participate in mucosal defense against the adhesive mouse pathogen *Citrobacter rodentium* [16] as well as against dextran sulfate sodium-induced colitis with increased non-specific bacterial invasion into the mouse mucosa as a model of ulcerative colitis [18]. Using our established animal model of *E. coli* O157:H7 infection [23] we have shown, in the current study, the importance of cathelicidin in mucosal protection against this important human pathogen. In contrast to our previous experiments on C57BL/6 mice [23] the mouse strain 129/SvJ appears to be more resistant to *E. coli* O157:H7 infection. This may be explained by increased resistance to Stx observed by others [40]. Deficiency in cathelicidin, however, led to a significantly increased bacterial colonization, increased invasion of intestinal mucus and formation of attaching and effacing lesions typical for *E. coli* O157:H7 colitis. Moreover, while cathelicidin-producing mice remained completely asymptomatic during 14 days after inoculation with the pathogenic bacteria, 80% of cathelicidin-deficient mice became symptomatic. Systemic symptoms and signs seemed to develop as a consequence of Stx release since only Stx2-positive *E. coli* O157:H7 were able to induce disease. The distribution of the Stx receptor Gb3 in mice is different than in humans [6] explaining differences between the human and murine disease. Intraperitoneal injection with purified Stx2 did not have a different effect on the cathelicidin-producing and deficient animals confirming that the level of cathelicidin protection is located predominantly in the intestinal mucosa.

In our study, we focused on infection with *E. coli* O157:H7 because of its clinical relevance. In up to 15% of cases, *E. coli* O157:H7 infection leads to hemolytic uremic syndrome, a potentially life-threatening and debilitating disease, which is considered to be the most common cause of acute renal failure in small children in the western world. There is no specific treatment for this disease. The use of antibiotics during the diarrheal phase of infection may increase toxin release and the occurrence of complications and is therefore not recommended [41]. Novel strategies are being designed to prevent or treat *E. coli* O157:H7-mediated disease, including Stx-component vaccines, toxin neutralizers, small molecules that block the toxic effects of Stx [42], [43] and monoclonal antibodies blocking the terminal complement cascade [44]. The narrow therapeutic window, however, makes treatment trials challenging. Our results bring a new possibility for the prevention or treatment of the disease. Administration of cathelicidin or stimulation of endogenous production of the peptide may prove to be a new treatment strategy. There are already several possibilities to stimulate cathelcidin. In different settings, vitamin D, zinc, butyrate and phenylbutyrate have been described as strong inducers of peptide production [45], [46], [47], [48], [49], [50].

In conclusion, we described for the first time the relevance of cathelicidin production in the colon for the protection against the important human pathogen *E. coli* O157:H7. Our experiments elucidated the mechanism of this protective effect of the peptide by influencing the mechanical and antimicrobial barrier in the intestinal mucosa. Our results may lead to a better understanding of mucosal immunity in the gut as well as to development of new treatment strategies for *E. coli* O157:H7-mediated diseases.

## References

[1] Griffin PM, Ostroff SM, Tauxe RV, Greene KD, Wells JG, et al (1988) Illnesses associated with *Escherichia coli* O157:H7 infections. A broad clinical spectrum. Ann Intern Med 109: 705–712. 3056169 10.7326/0003-4819-109-9-705

[2] Jerse AE, Yu J, Tall BD, Kaper JB (1990) A genetic locus of enteropathogenic *Escherichia coli* necessary for the production of attaching and effacing lesions on tissue culture cells. Proc Natl Acad Sci U S A 87: 7839–7843. 2172966 10.1073/pnas.87.20.7839 PMC54845

[3] Nataro JP, Kaper JB (1998) Diarrheagenic *Escherichia coli* . Clin Microbiol Rev 11: 142–201. 9457432 10.1128/cmr.11.1.142 PMC121379

[4] Karpman D, Sartz L, Johnson S (2010) Pathophysiology of typical hemolytic uremic syndrome. Semin Thromb Hemost 36: 575–585. 20865634 10.1055/s-0030-1262879

[5] Ståhl AL, Sartz L, Nelsson A, Bekassy ZD, Karpman D (2009) Shiga toxin and lipopolysaccharide induce platelet-leukocyte aggregates and tissue factor release, a thrombotic mechanism in hemolytic uremic syndrome. PLoS One 4: e6990. 19750223 10.1371/journal.pone.0006990 PMC2735777

[6] Obrig TG, Karpman D (2011) Shiga toxin pathogenesis: Kidney complications and renal failure. Curr Top Microbiol Immunol 10.1007/82_2011_172 PMC3779650 21983749

[7] Pennington H (2010) *Escherichia coli* O157. Lancet 376: 1428–1435. 20971366 10.1016/S0140-6736(10)60963-4

[8] Zasloff M (2002) Antimicrobial peptides of multicellular organisms. Nature 415: 389–395. 11807545 10.1038/415389a

[9] Gudmundsson GH, Agerberth B, Odeberg J, Bergman T, Olsson B, et al (1996) The human gene FALL39 and processing of the cathelin precursor to the antibacterial peptide LL-37 in granulocytes. Eur J Biochem 238: 325–332. 8681941 10.1111/j.1432-1033.1996.0325z.x

[10] Gallo RL, Kim KJ, Bernfield M, Kozak CA, Zanetti M, et al (1997) Identification of CRAMP, a cathelin-related antimicrobial peptide expressed in the embryonic and adult mouse. J Biol Chem 272: 13088–13093. 9148921 10.1074/jbc.272.20.13088

[11] Kai-Larsen Y, Luthje P, Chromek M, Peters V, Wang X, et al (2010) Uropathogenic *Escherichia coli* modulates immune responses and its curli fimbriae interact with the antimicrobial peptide LL-37. PLoS Pathog 6: e1001010. 20661475 10.1371/journal.ppat.1001010 PMC2908543

[12] Overhage J, Campisano A, Bains M, Torfs EC, Rehm BH, et al (2008) Human host defense peptide LL-37 prevents bacterial biofilm formation. Infect Immun 76: 4176–4182. 18591225 10.1128/IAI.00318-08 PMC2519444

[13] Kin NW, Chen Y, Stefanov EK, Gallo RL, Kearney JF (2011) Cathelin-related antimicrobial peptide differentially regulates T- and B-cell function. Eur J Immunol 41: 3006–3016. 21773974 10.1002/eji.201141606 PMC3234162

[14] Nizet V, Ohtake T, Lauth X, Trowbridge J, Rudisill J, et al (2001) Innate antimicrobial peptide protects the skin from invasive bacterial infection. Nature 414: 454–457. 11719807 10.1038/35106587

[15] Chromek M, Slamova Z, Bergman P, Kovacs L, Podracka L, et al (2006) The antimicrobial peptide cathelicidin protects the urinary tract against invasive bacterial infection. Nat Med 12: 636–641. 16751768 10.1038/nm1407

[16] Iimura M, Gallo RL, Hase K, Miyamoto Y, Eckmann L, et al (2005) Cathelicidin mediates innate intestinal defense against colonization with epithelial adherent bacterial pathogens. J Immunol 174: 4901–4907. 15814717 10.4049/jimmunol.174.8.4901

[17] Hase K, Eckmann L, Leopard JD, Varki N, Kagnoff MF (2002) Cell differentiation is a key determinant of cathelicidin LL-37/human cationic antimicrobial protein 18 expression by human colon epithelium. Infect Immun 70: 953–963. 11796631 10.1128/iai.70.2.953-963.2002 PMC127717

[18] Koon HW, Shih DQ, Chen J, Bakirtzi K, Hing TC, et al (2011) Cathelicidin Signaling via the Toll-like Receptor Protects Against Colitis in Mice. Gastroenterology 141: 1852–1863 e1853. 21762664 10.1053/j.gastro.2011.06.079 PMC3199285

[19] Islam D, Bandholtz L, Nilsson J, Wigzell H, Christensson B, et al (2001) Downregulation of bactericidal peptides in enteric infections: a novel immune escape mechanism with bacterial DNA as a potential regulator. Nat Med 7: 180–185. 11175848 10.1038/84627

[20] O'Brien AD, Melton AR, Schmitt CK, McKee ML, Batts ML, et al (1993) Profile of *Escherichia coli* O157:H7 pathogen responsible for hamburger-borne outbreak of hemorrhagic colitis and hemolytic uremic syndrome in Washington. J Clin Microbiol 31: 2799–2801. 8253989 10.1128/jcm.31.10.2799-2801.1993 PMC266020

[21] Karpman D, Connell H, Svensson M, Scheutz F, Alm P, et al (1997) The role of lipopolysaccharide and Shiga-like toxin in a mouse model of *Escherichia coli* O157:H7 infection. J Infect Dis 175: 611–620. 9041333 10.1093/infdis/175.3.611

[22] Bekassy ZD, Calderon Toledo C, Leoj G, Kristoffersson A, Leopold SR, et al (2011) Intestinal damage in enterohemorrhagic *Escherichia coli* infection. Pediatr Nephrol 26: 2059–2071. 20809220 10.1007/s00467-010-1616-9

[23] Calderon Toledo C, Rogers TJ, Svensson M, Tati R, Fischer H, et al (2008) Shiga toxin-mediated disease in MyD88-deficient mice infected with *Escherichia coli* O157:H7. Am J Pathol 173: 1428–1439. 18832584 10.2353/ajpath.2008.071218 PMC2570133

[24] Johansson ME, Larsson JM, Hansson GC (2011) The two mucus layers of colon are organized by the MUC2 mucin, whereas the outer layer is a legislator of host-microbial interactions. Proc Natl Acad Sci U S A 108 Suppl 1: 4659–4665. 20615996 10.1073/pnas.1006451107 PMC3063600

[25] Thompson KL, Applegate TJ (2006) Feed withdrawal alters small-intestinal morphology and mucus of broilers. Poult Sci 85: 1535–1540. 16977838 10.1093/ps/85.9.1535

[26] Tai EK, Wong HP, Lam EK, Wu WK, Yu L, et al (2008) Cathelicidin stimulates colonic mucus synthesis by up-regulating MUC1 and MUC2 expression through a mitogen-activated protein kinase pathway. J Cell Biochem 104: 251–258. 18059019 10.1002/jcb.21615

[27] Frankel G, Phillips AD, Rosenshine I, Dougan G, Kaper JB, et al (1998) Enteropathogenic and enterohaemorrhagic *Escherichia coli*: more subversive elements. Mol Microbiol 30: 911–921. 9988469 10.1046/j.1365-2958.1998.01144.x

[28] Savage DC (1977) Microbial ecology of the gastrointestinal tract. Annu Rev Microbiol 31: 107–133. 334036 10.1146/annurev.mi.31.100177.000543

[29] Backhed F, Ley RE, Sonnenburg JL, Peterson DA, Gordon JI (2005) Host-bacterial mutualism in the human intestine. Science 307: 1915–1920. 15790844 10.1126/science.1104816

[30] Louis P, Scott KP, Duncan SH, Flint HJ (2007) Understanding the effects of diet on bacterial metabolism in the large intestine. J Appl Microbiol 102: 1197–1208. 17448155 10.1111/j.1365-2672.2007.03322.x

[31] Schauber J, Svanholm C, Termen S, Iffland K, Menzel T, et al (2003) Expression of the cathelicidin LL-37 is modulated by short chain fatty acids in colonocytes: relevance of signalling pathways. Gut 52: 735–741. 12692061 10.1136/gut.52.5.735 PMC1773650

[32] Kankainen M, Paulin L, Tynkkynen S, von Ossowski I, Reunanen J, et al (2009) Comparative genomic analysis of *Lactobacillus rhamnosus* GG reveals pili containing a human- mucus binding protein. Proc Natl Acad Sci U S A 106: 17193–17198. 19805152 10.1073/pnas.0908876106 PMC2746127

[33] Bishop JR, Gagneux P (2007) Evolution of carbohydrate antigens–microbial forces shaping host glycomes? Glycobiology 17: 23R–34R. 10.1093/glycob/cwm005 17237137

[34] Johansson ME, Phillipson M, Petersson J, Velcich A, Holm L, et al (2008) The inner of the two Muc2 mucin-dependent mucus layers in colon is devoid of bacteria. Proc Natl Acad Sci U S A 105: 15064–15069. 18806221 10.1073/pnas.0803124105 PMC2567493

[35] Otte JM, Zdebik AE, Brand S, Chromik AM, Strauss S, et al (2009) Effects of the cathelicidin LL-37 on intestinal epithelial barrier integrity. Regul Pept 156: 104–117. 19328825 10.1016/j.regpep.2009.03.009

[36] Jager S, Stange EF, Wehkamp J (2010) Antimicrobial peptides in gastrointestinal inflammation. Int J Inflam 2010: 910283. 21151692 10.4061/2010/910283 PMC2992817

[37] Meyer-Hoffert U, Hornef MW, Henriques-Normark B, Axelsson LG, Midtvedt T, et al (2008) Secreted enteric antimicrobial activity localises to the mucus surface layer. Gut 57: 764–771. 18250125 10.1136/gut.2007.141481

[38] Wilson CL, Ouellette AJ, Satchell DP, Ayabe T, Lopez-Boado YS, et al (1999) Regulation of intestinal alpha-defensin activation by the metalloproteinase matrilysin in innate host defense. Science 286: 113–117. 10506557 10.1126/science.286.5437.113

[39] Salzman NH, Ghosh D, Huttner KM, Paterson Y, Bevins CL (2003) Protection against enteric salmonellosis in transgenic mice expressing a human intestinal defensin. Nature 422: 522–526. 12660734 10.1038/nature01520

[40] Motto DG, Chauhan AK, Zhu G, Homeister J, Lamb CB, et al (2005) Shigatoxin triggers thrombotic thrombocytopenic purpura in genetically susceptible ADAMTS13-deficient mice. J Clin Invest 115: 2752–2761. 16200209 10.1172/JCI26007 PMC1240119

[41] Wong CS, Jelacic S, Habeeb RL, Watkins SL, Tarr PI (2000) The risk of the hemolytic-uremic syndrome after antibiotic treatment of *Escherichia coli* O157:H7 infections. N Engl J Med 342: 1930–1936. 10874060 10.1056/NEJM200006293422601 PMC3659814

[42] Bitzan M (2009) Treatment options for HUS secondary to *Escherichia coli* O157:H7. Kidney Int Suppl: S62–66. 10.1038/ki.2008.624 19180140

[43] Mukhopadhyay S, Linstedt AD (2012) Manganese blocks intracellular trafficking of Shiga toxin and protects against Shiga toxicosis. Science 335: 332–335. 22267811 10.1126/science.1215930 PMC5367627

[44] Lapeyraque AL, Malina M, Fremeaux-Bacchi V, Boppel T, Kirschfink M, et al (2011) Eculizumab in severe Shiga-toxin-associated HUS. N Engl J Med 364: 2561–2563. 21612462 10.1056/NEJMc1100859

[45] Hertting O, Holm A, Luthje P, Brauner H, Dyrdak R, et al (2010) Vitamin D induction of the human antimicrobial peptide cathelicidin in the urinary bladder. PLoS One 5: e15580. 21179490 10.1371/journal.pone.0015580 PMC3001888

[46] Raqib R, Sarker P, Bergman P, Ara G, Lindh M, et al (2006) Improved outcome in shigellosis associated with butyrate induction of an endogenous peptide antibiotic. Proc Natl Acad Sci U S A 103: 9178–9183. 16740661 10.1073/pnas.0602888103 PMC1482586

[47] Talukder P, Satho T, Irie K, Sharmin T, Hamady D, et al (2011) Trace metal zinc stimulates secretion of antimicrobial peptide LL-37 from Caco-2 cells through ERK and p38 MAP kinase. Int Immunopharmacol 11: 141–144. 21035435 10.1016/j.intimp.2010.10.010

[48] Wang TT, Nestel FP, Bourdeau V, Nagai Y, Wang Q, et al (2004) Cutting edge: 1,25-dihydroxyvitamin D3 is a direct inducer of antimicrobial peptide gene expression. J Immunol 173: 2909–2912. 15322146 10.4049/jimmunol.173.5.2909

[49] Sarker P, Ahmed S, Tiash S, Rekha RS, Stromberg R, et al (2011) Phenylbutyrate counteracts Shigella mediated downregulation of cathelicidin in rabbit lung and intestinal epithelia: a potential therapeutic strategy. PLoS One 6: e20637. 21673991 10.1371/journal.pone.0020637 PMC3108617

[50] Liu PT, Stenger S, Li H, Wenzel L, Tan BH, et al (2006) Toll-like receptor triggering of a vitamin D-mediated human antimicrobial response. Science 311: 1770–1773. 16497887 10.1126/science.1123933

